# Determining glycosyltransferase functional order via lethality due to accumulated O-antigen intermediates, exemplified with *Shigella flexneri* O-antigen biosynthesis

**DOI:** 10.1128/aem.02203-23

**Published:** 2024-05-15

**Authors:** Jilong Qin, Yaoqin Hong, Makrina Totsika

**Affiliations:** 1 Centre for Immunology and Infection Control, School of Biomedical Sciences, Queensland University of Technology, Brisbane, Queensland, Australia; 2 Max Planck Queensland Centre, Queensland University of Technology, Brisbane City, Queensland, Australia; Washington University in St. Louis, St. Louis, Missouri, USA

**Keywords:** *Shigella flexneri*, O antigen, glycosyltransferase, UndP sequestration, lipopolysaccharide, Gram-negative pathogen

## Abstract

The O antigen (OAg) polysaccharide is one of the most diverse surface molecules of Gram-negative bacterial pathogens. The structural classification of OAg, based on serological typing and sequence analysis, is important in epidemiology and the surveillance of outbreaks of bacterial infections. Despite the diverse chemical structures of OAg repeating units (RUs), the genetic basis of RU assembly remains poorly understood and represents a major limitation in assigning gene functions in polysaccharide biosynthesis. Here, we describe a genetic approach to interrogate the functional order of glycosyltransferases (GTs). Using *Shigella flexneri* as a model, we established an initial glycosyltransferase (IT)-controlled system, which allows functional order allocation of the subsequent GT in a 2-fold manner as follows: (i) first, by reporting the growth defects caused by the sequestration of UndP through disruption of late GTs and (ii) second, by comparing the molecular sizes of stalled OAg intermediates when each putative GT is disrupted. Using this approach, we demonstrate that for RfbF and RfbG, the GT involved in the assembly of *S. flexneri* backbone OAg RU, RfbG, is responsible for both the committed step of OAg synthesis and the third transferase for the second L-Rha. We also show that RfbF functions as the last GT to complete the *S. flexneri* OAg RU backbone. We propose that this simple and effective genetic approach can be also extended to define the functional order of enzymatic synthesis of other diverse polysaccharides produced both by Gram-negative and Gram-positive bacteria.

**IMPORTANCE:**

The genetic basis of enzymatic assembly of structurally diverse O antigen (OAg) repeating units (RUs) in Gram-negative pathogens is poorly understood, representing a major limitation in our understanding of gene functions for the synthesis of bacterial polysaccharides. We present a simple genetic approach to confidently assign glycosyltransferase (GT) functions and the order in which they act during assembly of the OAg RU. We employed this approach to determine the functional order of GTs involved in *Shigella flexneri* OAg assembly. This approach can be generally applied in interrogating GT functions encoded by other bacterial polysaccharides to advance our understanding of diverse gene functions in the biosynthesis of polysaccharides, key knowledge in advancing biosynthetic polysaccharide production.

## INTRODUCTION

The O antigen (OAg) is a polysaccharide made of oligosaccharide repeating units (RUs) that form part of the Gram-negative bacterial surface lipopolysaccharide (LPS). Bacterial OAg is an important virulence factor, as its loss renders many Gram-negative bacteria sensitive to host antimicrobials (1–3) and attenuates colonization of host niches (4). Surface OAg structures are also major targets for host niche-residing bacteriophages and the host immune system due to their high immunogenicity. Moreover, OAg are major glycans directly contributing to host cell attachment through specific glycan:glycan interactions (5, 6) and indirectly contributing to pathogenesis by modulating the exposure of other virulence factors (7, 8), hence are constantly under strong selection pressure. These factors are thought to be evolutionary drivers of high OAg diversity, giving rise to over 180 forms of OAg structures characterized so far solely in *Escherichia coli* (including *Shigella* strains) (9), representing one of the most variable bacterial cell surface components. The variability of OAg is due to not only the different saccharides present in the OAg RU but also the linkages within and between the RU (9), which are catalyzed by a variety of glycosyltransferases (GTs) and polymerases. OAg variability was therefore used as the basis of serotyping schemes (10) applied widely to Gram-negative bacteria and is of major epidemiological importance (11).

Genes for the biogenesis of OAg are clustered in a locus flanked by the *galF* and *gnd* genes in the majority of *E. coli* genomes (9). The O locus encodes proteins responsible for (i) the biosynthesis of nucleotide diphospho (NDP) sugars as OAg RU precursors, (ii) GTs for the assembly of OAg RU, and (iii) RU polymerization and translocation. Genes for the synthesis of NDP sugars, RU polymerization, and translocation are generally identifiable by sequence alone, due to conservation in their primary sequence, secondary structure, and protein function, which assigns them to biochemically well-characterized protein families (12). In contrast, the genes for GTs, although often identified by sequence similarity alone, are difficult to functionally assign to specific sugar linkages with confidence (13, 14). In some occasions, GT gene function can be predicted with confidence by combining sequence information with information from the OAg chemical structure (2). However, OAg structures with linkages between multiple saccharides that are identical provide limited guidance in accurately allocating GT functions, representing a major limitation in assigning gene functions for bacterial polysaccharides.

**Fig 1:**
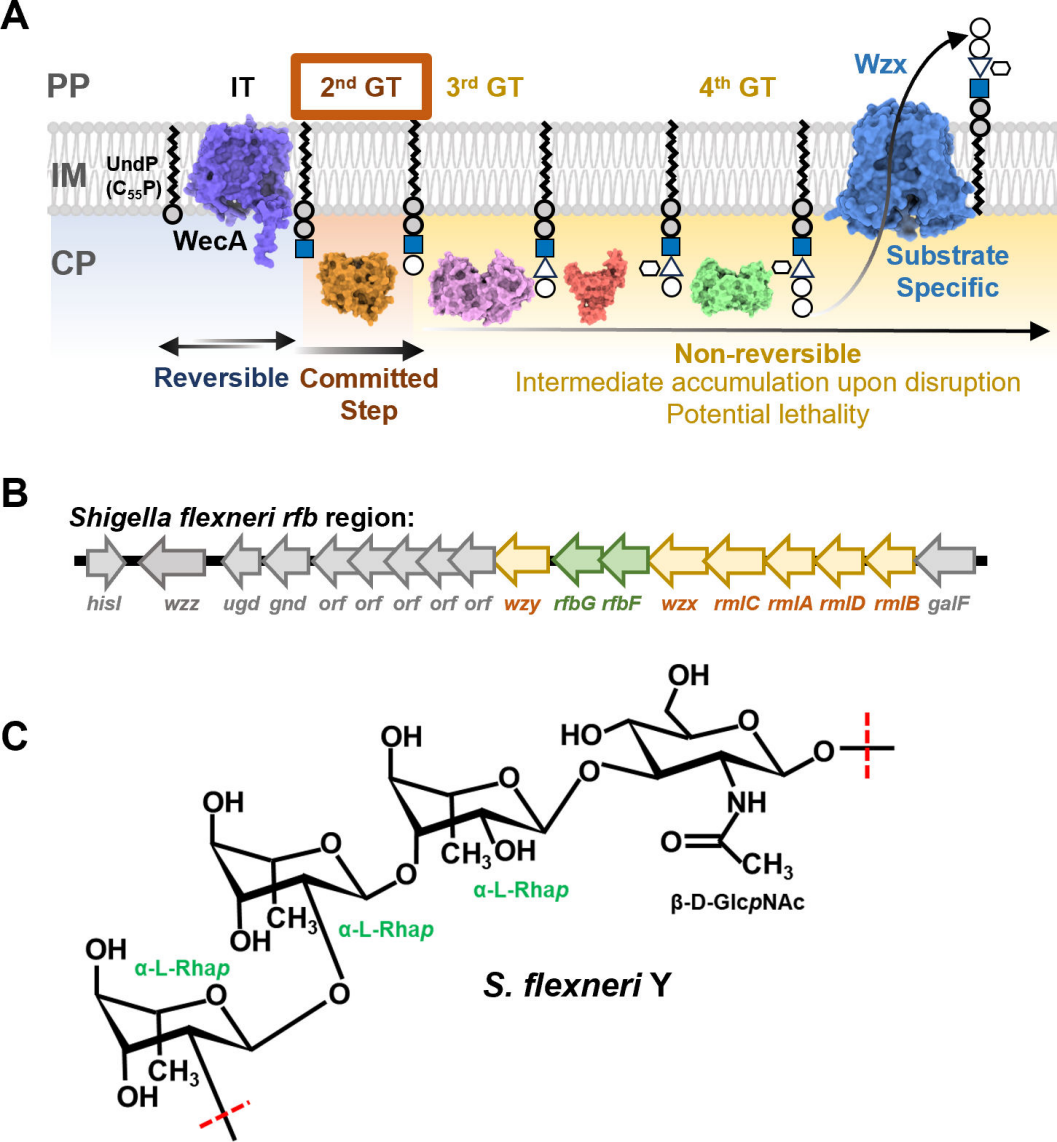
OAg RU biogenesis and *Shigella flexneri* Y OAg structure. (**A**) Stringency of OAg RU synthesis pathways exemplified with K-12 O16 OAg biogenesis. The incomplete OAg RU intermediates on the lipid carrier UndP are not the preferred substrate of Wzx and will remain in the cytosolic face until the complete RU is synthesized. The transfer of saccharide by the second GT is the committed step of OAg biogenesis, and disruptions beyond the second GT including wzx will cause growth defects due sequestration of UndP. PP, periplasm, IM, inner membrane, CP, cytoplasm, IT, initial glycosyltransferase, GT, glycosyltransferase. (**B**) Schematic representation of *S. flexneri rfb* region. Genes encoding for GTs RfbF and RfbG are highlighted in green. (**C**) The chemical structure of *S. flexneri* Y serotype OAg (25).

The majority (93%) of OAg in *E. coli* are synthesized by the Wzx/Wzy-dependent pathway (Fig. 1A) (9). N-acetylglucosamine (GlcNAc) is the initial sugar that is transferred onto the lipid carrier undecaprenol phosphate (UndP/C_55_-P) by the initial glycosyltransferase (IT) WecA (15). The *wecA* gene is located in the gene cluster for the biosynthesis of enterobacterial common antigen (ECA). The GlcNAc-phosphotransferase reaction by the IT WecA is reported to be reversible (Fig. 1A), and the resulting product UndPP-GlcNAc is also the substrate for the second GT (second GT) in the ECA biogenesis (16). In contrast, the the second GT reaction in OAg biogenesis, which adds the second sugar onto UndPP-GlcNAc, is non-reversible and therefore is the committed step in OAg biosynthesis (Fig. 1A). Owing to the specificity of GTs for their lipid-linked acceptor, the complete OAg RU is synthesized in a strict sequential manner to then be flipped across the inner membrane (IM) by the flippase Wzx (Fig. 1A) (17, 18). Wzx has specificity toward OAg RU, in that only the complete RU is efficiently flipped across the IM (Fig. 1A) (19, 20). The specificities of GTs and Wzx are the key characteristics, ensuring correct cell surface presentation of OAg. This is because the ligase WaaL, which ligates OAg onto the lipid-A core forming Smooth LPS (S-LPS), lacks specificity toward its donors (21). Disruption of OAg sugar precursor biosynthesis, GTs and Wzx, will stall the OAg biogenesis (17, 20, 22). However, there is no feedback mechanism to control OAg biosynthesis when the cytosolic steps beyond the committed step (i.e. steps catalyzed by late GTs and the flipping step catalyzed by Wzx) are disrupted. Indeed, cells with such disruptions accumulate a significant amount of UndPP-linked OAg intermediates and suffer severe growth stress creating lethal phenotypes (Fig. 1A) (17, 19, 23). UndP employed in the OAg synthesis pathways is only released by Wzy and WaaL in the periplasm (PP) and then recycled back to cytosolic side of the IM, a process that is vital to the biogenesis of other cell envelope components especially for peptidoglycan (PG), an essential synthesis pathway for cell viability (24). However, the UndP in the stalled UndPP-linked OAg intermediates in the cytosolic side of the IM is inaccessible by Wzy and WaaL, thereby causing UndP sequestration leading to growth defects (19). Here, we demonstrate that the lack of a feedback-control mechanism could be exploited experimentally as a simple and effective strategy to determine the functional order of GTs in OAg biosynthesis. In particular, the determination of the second GT in the gene cluster, whereby only the disruption of late GTs as well as Wzx, but not the committed step catalyzed by the second GT, would accumulate OAg intermediates and generate lethal phenotypes.

We use the OAg biosynthesis pathway in *S. flexneri* Y serotype as a proof of principle for a genetic approach that can confidently identify the second GT in the *rfb* locus, in a system where the expression of IT is tightly controlled. The *rfb* region of *S. flexneri* Y is sufficiently responsible for the synthesis and assembly of the Y serotype OAg (26) (Fig. 1B), which has the chemically defined RU structure →2)-α-L-Rha*p*III-(1→2)-α-L-Rha*p*II-(1→3)-α-L-Rha*p*I-(1→3)-β-D-Glc*p*NAc-(1→ (25) (Fig. 1C) and is shared by all *S. flexneri* serotype strains as the backbone structure. However, only two genes (*rfbF* and *rfbG,* also referred as *wbgF* and *wbgG*, respectively, according to the bacterial polysaccharide gene nomenclature (BPGN) scheme (27, 28)) are predicted to be rhamnose GTs in the *rfb* region (Fig. 1B). It is therefore difficult to allocate RfbF- and RfbG-specific functions in *S. flexneri* Y OAg sugar linkages. Here, we demonstrate that disruption of RfbF as well as Wzx, but not RfbG, generated lethal phenotypes and confirmed the role of RfbG as the second GT in *S. flexneri* OAg synthesis. By comparing the molecular sizes of the small dead-end intermediates that were capped onto LPS molecules in strains with lethality, we were also able to determine that RfbG is responsible for the transfer of the first two rhamnoses, whereas RfbF is responsible for the last rhamnose. This is the first study to assign OAg GT functional order for a *S. flexneri Y* strain, demonstrated as an example of the allocation of functional order for polysaccharide GTs via a genetic approach.

## RESULTS

### RfbG is the second GT in the assembly of *S. flexneri* Y OAg

**Fig 2:**
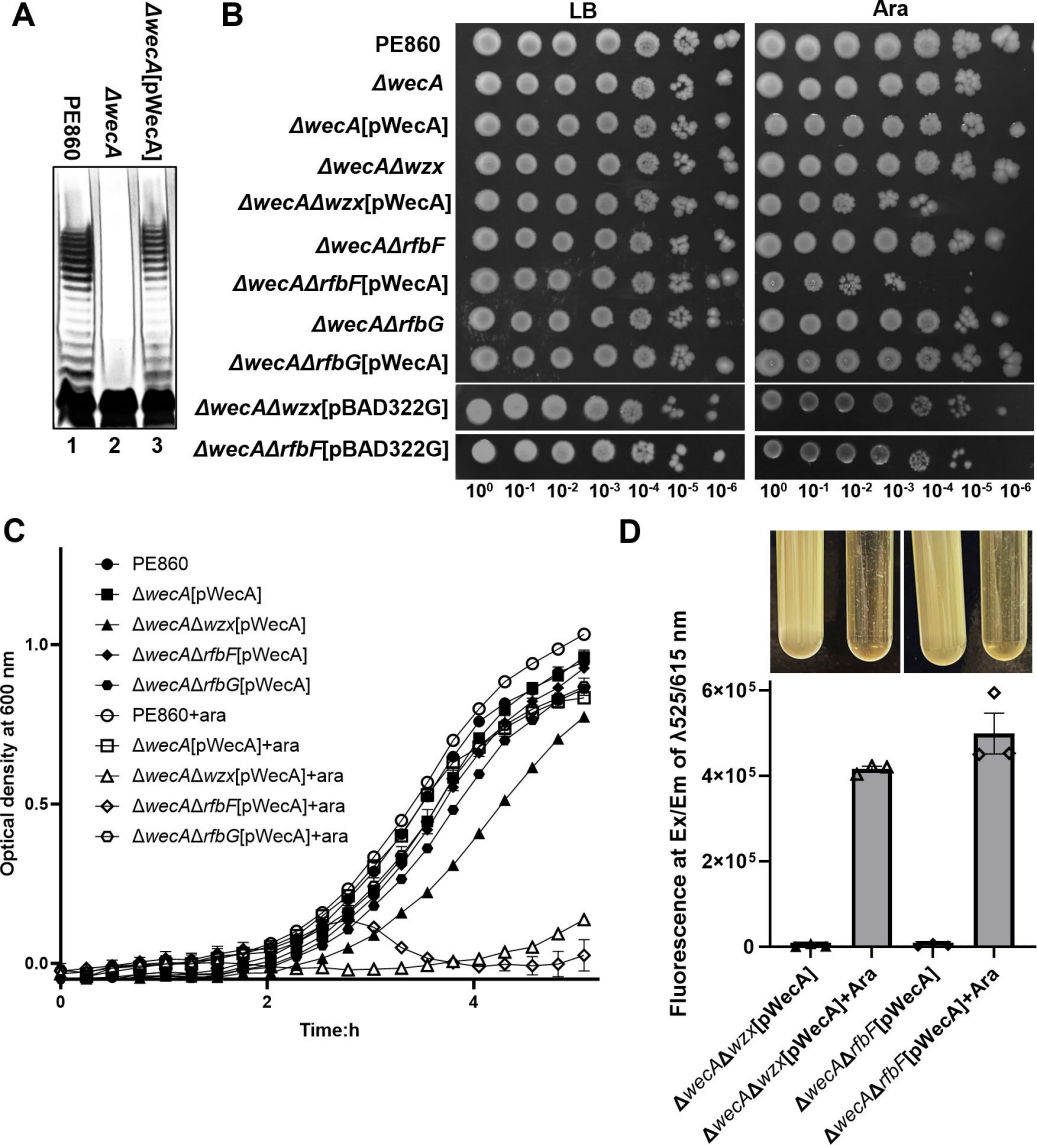
Lethality caused by OAg intermediate accumulation in *S. flexneri* Y serotype strains with disruptions in late GTs and translocase wzx. (**A**) LPS silver staining of LPS samples prepared from indicated *S. flexneri* strains. (**B**) Viability of indicated bacterial strains in 10-fold serial dilutions in the presence or absence of cloned gene induction. (**C**) Growth curves of indicated *S. flenxeri* strains in LB without or with 10 mM arabinose for pWecA induction. (**D**) Release of cellular DNA of indicated *S. flexneri* strains in culture supernatant upon pWecA induction. Cell lysis was imaged as loss of turbidity in culture media. Data represent three biological repeats.

As a first step, we deleted *wecA* encoding for the OAg IT in *S. flexneri* strain PE860 and abolished the production of S-LPS in this strain (Fig. 2A, lane 2). This was done to avoid potential deleterious effects of subsequent mutations introduced in the OAg late GTs, which may lead to genetic instability and secondary suppressor mutations that would confound the interpretation of results in our study. We then engineered a WecA expression construct under *pBAD* promoter control, the expression of which could complement the Δ*wecA* mutant to produce S-LPS similar to that of WT PE860 (Fig. 2A, lane 1) upon arabinose induction (Fig. 2A, lane 3). We first confirmed that the induction of WecA in the Δ*wecA* mutant background did not have any observable effect on cell viability (Fig. 2B) or growth kinetics (Fig. 2C). We then successfully deleted the *wzx* flippase gene in the Δ*wecA* background with no impact on cell viability (Fig. 2B). In contrast, initiation of OAg RU synthesis by induction of WecA in Δ*wecA*Δ*wzx* severely affected cell viability (Fig. 2B) and caused cells lysis and release of cellular DNA when growing in liquid media (Fig. 2C and D). This is consistent with the results reported for a *Salmonella enterica* Δ*galE*Δ*wzx* mutant with OAg production under exogenous galactose control, showing that Wzx is essential when the OAg RU is assembled (23). These results confirmed that the sequestration of UndP by OAg dead-end intermediates affects cell viability and growth, creating observable lethal phenotypes in systems with OAg production under tight control. We then deleted the two GTs *rfbF* and *rfbG*, respectively, in the Δ*wecA* background*,* with neither having any observable effect on cell viability (Fig. 2B). However, when OAg production was induced by *in trans* WecA expression, the Δ*wecA*Δ*rfbF* mutant but not the Δ*wecA*Δ*rfbG* showed notable defects in cell viability and increased cell lysis (Fig. 2B through D), similar to that observed with Δ*wecA*Δ*wzx* mutant expressing WecA. Disruption of GTs acting directly after WecA in OAg assembly process (the committed step) will prevent UndP from entering disrupted OAg synthesis pathways and abolish the sequestering effect, as the product of WecA UndPP-GlcNAc will be redirected into making ECA and the surplus amounts of GlcNAc dissociate from UndP by WecA as discussed previously. Therefore, our results suggest that (i) RfbG is the second GT catalyzing the committed step for *S. flexneri* Y OAg RU assembly and (ii) RfbF acts after RfbG and, when disrupted, leads to accumulated intermediates that are not efficiently recognized and flipped by Wzx, thereby sequestering UndP in the OAg synthesis pathway and creating lethal phenotypes.

### RfbG cross-complements *E. coli* K-12 producing O16 OAg

**Fig 3:**
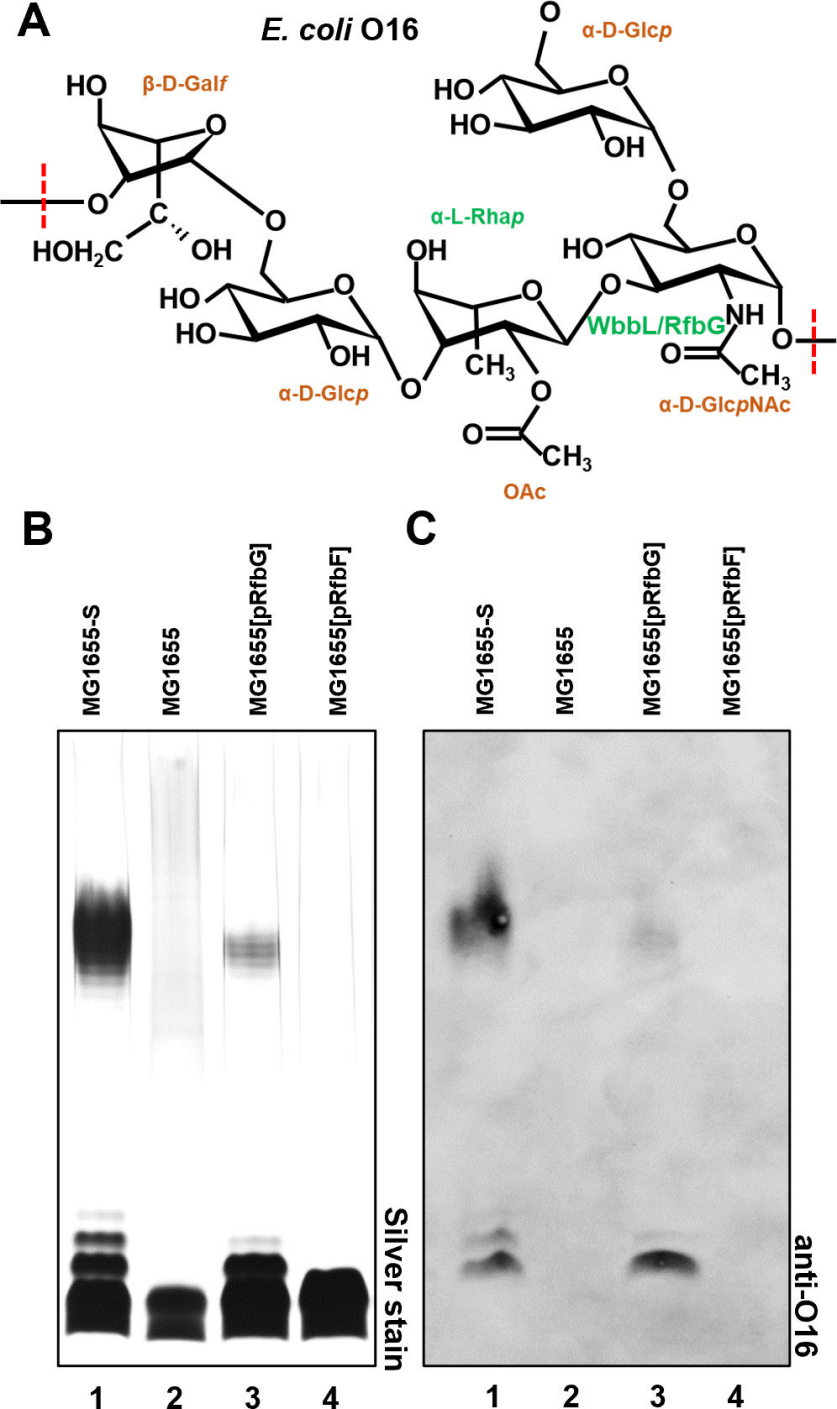
Cross-complementation of *E. coli* K-12 O16 OAg production by *S. flexneri* GTs. (**A**) Same glycosidic linkage catalyzed by WbbL and RfbG is shown with *E. coli* K-12 O16 OAg structure (30). (**B**) LPS silver staining of *E. coli* K-12 MG1655 complemented with *S. flexneri* GTs. (**C**) Western immunoblotting of samples of *S. flexneri* strains shown in (B) with anti-O16 antibodies.

*E. coli* K-12 strain MG1655 is devoid of OAg due to a characterized mutation in *wbbL* (29), which encodes the second GT responsible for the formation of L-Rha*p*(1→3) linkage in the O16 OAg backbone structure (→2)-β-D-Gal*f*-(1→6)-α-D-Glc*p*-(1→3)-α-L-Rha*p*-(1→3)-α-D-Glc*p*NAc-(1→) (Fig. 3A) (30). Repairing *wbbL* in this strain (designated as MG1655-S) (22) produced S-LPS (Fig. 3B, lane 1). The L-Rha*p*(1→3) linkage between O16 and *S. flexneri* Y (Fig. 1C) is identical; hence, to validate the GT function of RfbG, we expressed both RfbG and RfbF in MG1655, respectively. Indeed, expression of RfbG (Fig. 3B, lane 3), but not RfbF (Fig. 3B, lane 4), restored the production of S-LPS in MG1655, albeit at reduced levels in comparison to the *wbbL*-repaired MG1655-S strain (Fig. 3B, lane 1). Western immunoblotting with monospecific anti-O16 antiserum confirmed that the restored S-LPS is composed of O16 OAg (Fig. 3C, lane 3). Therefore, our results confirmed the role of RfbG as the second GT catalyzing L-Rha*p*(1→3) linkage in the *S. flexneri* Y OAg.

### RfbG is a dual L-Rha transferase and RfbF is the last GT for *S. flexneri* Y OAg synthesis

**Fig 4:**
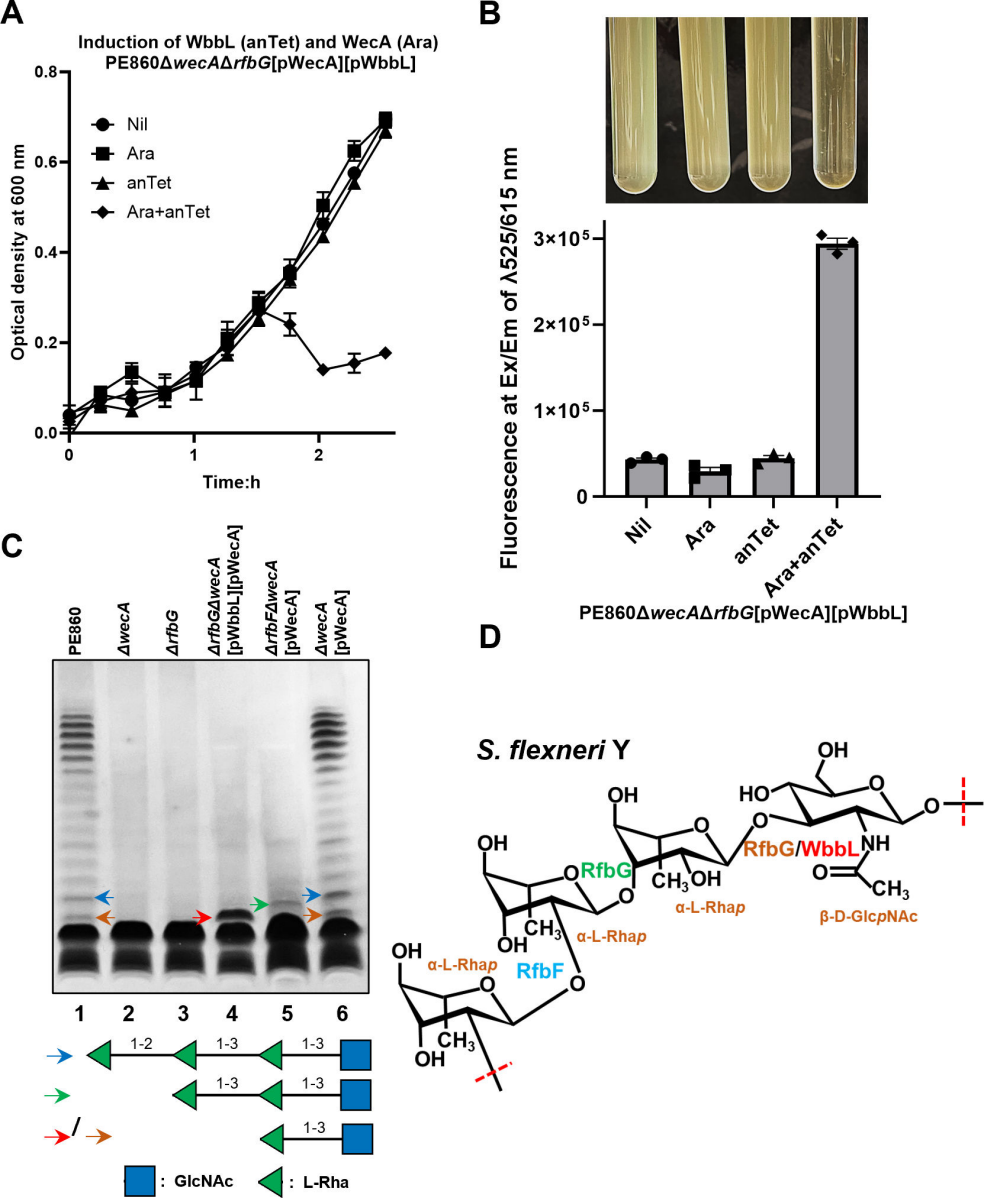
Ligation of incomplete *S. flexneri* OAg intermediates onto LPS in cells with lysis. (**A**) Growth curves of indicated *S. flexneri* strains with the expression of WecA non-induced or induced with 10 mM arabinose and/or WbbL non-induced or induced with 50 ng/mL anhydrotetracycline (anTet). (**B**) Release of cellular DNA of indicated *S. flexneri* strains in culture supernatant upon pWecA induction. Cell lysis was imaged as loss of turbidity in culture media. Data represent three biological repeats. (**C**) LPS silver staining of samples of indicated *S. flexneri* strains with early lysis. The predicted OAg saccharides capped onto LPS are marked by arrows and corresponding structures shown (GlcNAc-Rha in orange and red, GlcNAc-Rha-Rha in green, and GlcNAc-Rha-Rha-Rha in blue). (**D**) Proposed glycosidic linkages catalyzed by RfbG and RfbF shown in the *S. flexneri* Y OAg structure.

Since RfbG could functionally complement the *wbbL* mutation in *E. coli* K-12 restoring O16 OAg production, to further assign the function of RfbG and RfbF to the glycosidic linkages between the remaining two L-Rha in *S. flexneri* Y OAg, we then cross-complemented the PE860Δ*wecA*Δ*rfbG* mutant with plasmids expressing WecA and WbbL under the tight control of *pBAD* and *pTet* promoters, respectively. Interestingly, although induction of either *wecA* or *wbbL* alone in PE860Δ*wecA*Δ*rfbG* showed no growth impact in liquid media, induction of both simultaneously caused cell lysis (Fig. 4A through B). This was confirmed by an increase in the release of DNA in culture supernatant detected in PE860Δ*wecA*Δ*rfbG* when both *wecA* and *wbbL* were induced (Fig. 4B). This suggests that expression of both WecA and WbbL in PE860*ΔwecAΔrfbG* caused accumulation of OAg intermediates that could not be recognized and processed by the downstream RfbF, thereby sequestering UndP and triggering lethality.

The accumulation of lipid-linked OAg intermediates in the cytoplasmic side of IM is due to the specificity of Wzx, leading to deficient translocation across the physical barrier to reach WaaL in the periplasmic side of IM and be ligated onto lipid-A core to form semi-rough LPS (SR-LPS, lipid A-core with single OAg RU). However, we hypothesized that strains showing the cell lysis phenotype could also have a disrupted IM and small amounts of incomplete OAg RU intermediates could gain access to WaaL to form incomplete RU-LPS. Indeed, through the analysis of bacterial samples harvested 30 min after either WecA and/or WbbL induction via silver-stained SDS-PAGE, we were able to compare the molecular sizes of these OAg intermediates to SR-LPS by SDS-PAGE (Fig. 4C, lanes 1 and 6, blue arrow). In the absence of *wecA* or *rfbG*, only the band of lipid A-core can be detected (Fig. 4C, lanes 2–3), suggesting that the UndPP-GlcNAc was not sufficiently flipped by the Wzx of PE860. The expression of both WecA and WbbL in PE860*ΔwecAΔrfbG* showed an intermediate LPS (Fig. 4C, lane 4, red arrow) band in relatively high amount. The molecular size of this intermediate LPS molecule is larger than the R-LPS band shown in both PE860Δ*wecA* and PE860Δ*rfbG* but is smaller than the intermediate LPS band of the PE860Δ*wecA*Δ*rfbF* expressing WecA (Fig. 4C, lane 5, green arrow). These two intermediate LPS bands detected in PE860Δ*wecA*Δ*rfbG* expressing WecA and WbbL (Fig. 4C, lane 4, red arrow) and PE860Δ*wecA*Δ*rfbF* expressing WecA (Fig. 4C, lane 5, green arrow) were both smaller than the SR-LPS of the WT PE860 (Fig. 4C, lane 1, blue arrow) or PE860Δ*wecA* expressing WecA (Fig. 4C, lane 6, blue arrow), hence are the lipid A-core capped with different incomplete OAg RU. These data infer that the incomplete RU-LPS band detected in PE860Δ*wecA*Δ*rfbF* expressing WecA is lipid A-core-GlcNAc-Rha-Rha and that the PE860Δ*wecA*Δ*rfbG* expressing both WecA and WbbL produced lipid A-core-GlcNAc-Rha. Interestingly, in the WT PE860 (Fig. 2A, lane 1 and Fig. 4C, lane 1) and PE860Δ*wecA* expressing WecA (Fig. 2A, lane 3 and Fig. 4C, lane 6), an intermediate band having the same molecular size as that detected in PE860Δ*wecA*Δ*rfbG* expressing both WecA and WbbL was consistently detected. This intermediate band was shown to have a smaller molecular size than the SR-LPS produced in a *S. flexneri* Y *ΔwzyB* mutant previously (31). Similarly, an intermediate band in relatively low quantity correlating to the incomplete RU-linked LPS was also detected for *S. enterica* group B OAg-LPS (20). These results suggest that Wzx of *S. flexneri* Y could also inefficiently recognize and flip UndPP-GlcNAc-Rha across IM. Taken together, we propose that RfbG is both the second and third GT transferring the first two L-rhamnoses onto UndPP-GlcNAc, and RfbF is the last GT transferring the third L-rhamnose to synthesize the complete *S. flexneri* Y OAg RU (Fig. 4D).

## DISCUSSION

**Fig 5:**
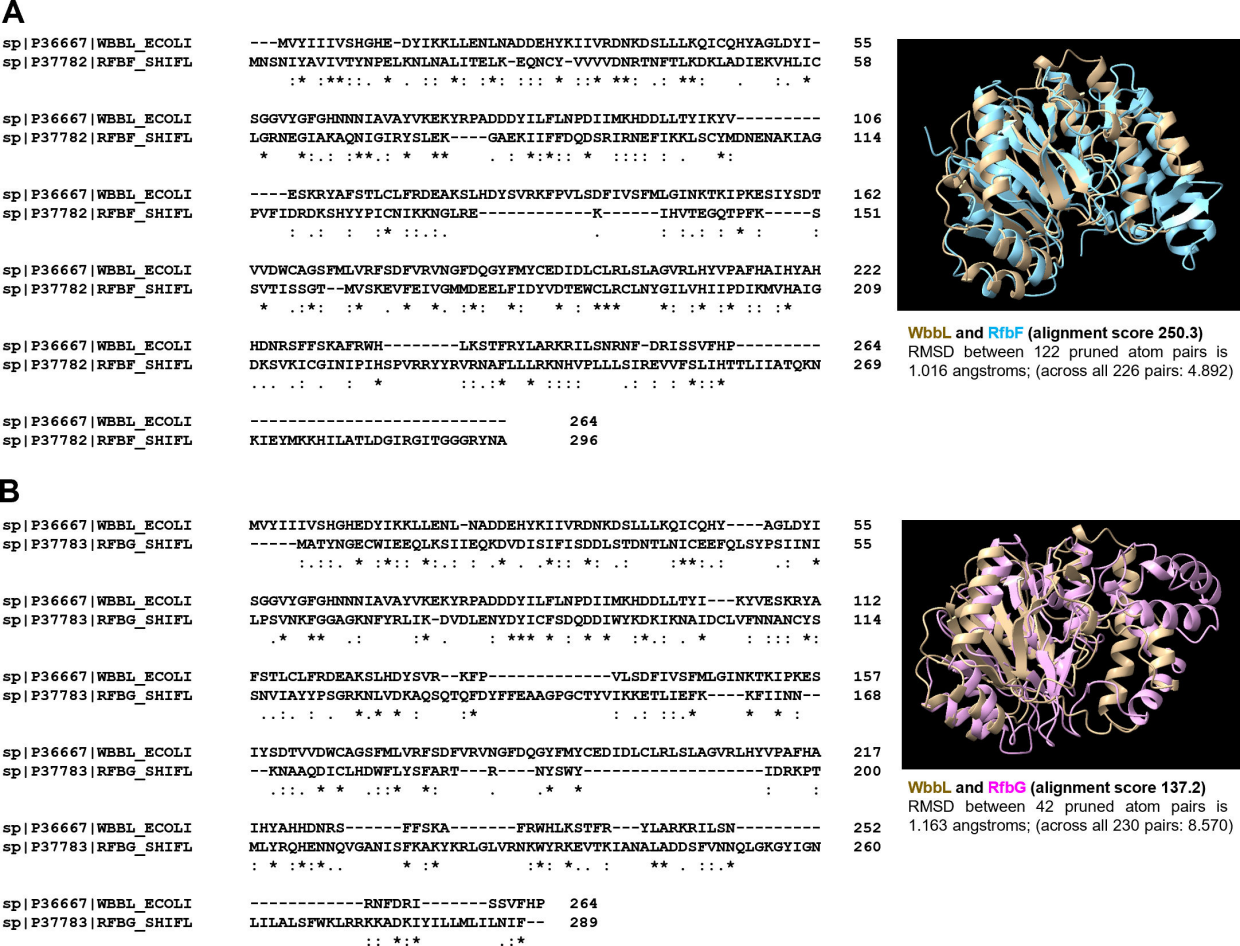
Protein sequence and structure comparison between *E. coli* K-12 WbbL and *S. flexneri* RfbF and RfbG. Protein sequence of WbbL was aligned with protein sequences of RfbF (**A**) and RfbG (**B**), respectively. The AlphaFold-predicted protein structure of WbbL (Brown) was superimposed with predicted structures of RfbF (blue) (**A**) and RfbG (pink) (**B**), respectively. The calculated overall structural alignment score, as well as the root mean square deviation (RMSD) of matched atom pairs, was reported.

The OAg component of LPS is highly immunogenic and is the target of humoral responses in the host. The highly diverse structures of OAg between and within species provide the basis for serological typing of Gram-negative bacterial pathogens and are important for epidemiology and surveillance of emerging pathogens. Despite advancements in OAg typing of pathogens via serology, or more recently whole genome sequence data analysis, in conjunction with over 180 solved OAg structures (9), determination of the genetic basis of RU assembly for the synthesis of different OAg structures remains a challenging task and represents a major hurdle in our understanding of gene functions for polysaccharide biosynthesis. This is because of the existence of numerous combinations of different donor sugars (at least 20 different sugars may compose the OAg), acceptor sugars/oligosaccharides, and sugar anomerism in polysaccharide structure. However, there is a lack of biochemical evidence to characterize all GT families found so far as both donor sugar in their NDP activated form and the lipid-linked incomplete OAg RU are not readily available. In addition, despite an overall low sequence similarity between functionally diverse GTs, they share remarkable structure similarities making it difficult to assign specific functions (32). For example, despite the poor protein sequence alignment of WbbL to RfbF (Fig. 5A) or RfbG (Fig. 5B), their AlphaFold-predicted (33) structures are relatively well aligned (Fig. 5). Interestingly, the predicted structure of RfbF (Fig. 5A), not RfbG, more closely matches that of WbbL (Fig. 5B), which could lead one to hypothesize that they are more likely to share similar function. However, we have shown here that RfbG, but not RfbF, can functionally complement the *wbbL::IS5* mutation in *E. coli* K-12 strain MG1655. This cautions that *in silico* analyses of GTs could lead to misleading assumptions and therefore highlights the usefulness of our method in experimentally providing GT functional order in bacteria.

Our study provides a simple method to effectively determine the GT functional order in the OAg RU assembly*,* which enabled us to successfully allocate functions to RfbG and RfbF in the assembly of *S. flexneri* backbone OAg. First, by comparing growth after mutating each of the putative GT genes in a system where the IT expression is tightly controlled, the committed step (second GT) of OAg RU assembly can be identified by having no growth impact when mutated. Second, in conjunction with the available chemical structure of the OAg RU, comparisons of the mass of the accumulated intermediates after mutating GTs individually allow us to aid the allocation of the function and the order of each GT on OAg RU assembly with confidence.

Our genetic approach for determining the committed GT function relies on the tight control of IT, a key strategy to avoid genetic instability when constructing GT deletion mutants. This is because the accumulation of dead-end intermediates via direct deletion of late GTs generates lethal conditions, potentially allowing a selection of suppressors preventing UndP entering the disrupted polysaccharide assembly pathways (34, 35). These phenomena may alter or complicate the interpretation of results. Therefore, we propose that when studying GT functions with late GT mutants, a parental strain with controlled IT or GT at the committed step expression should be used.

Disruption of the OAg production is a common strategy in constructing live attenuated vaccines. Our approach in determining the lethal phenotypes when deleting late GTs in the polysaccharide’s assembly pathways hence could provide important guidance in the construction of attenuated vaccine strains devoid of polysaccharide, in that the direct late GT mutations should be avoided to ensure the genetic stability of the live vaccine strain. It is interesting that a live attenuated *Shigella*-enterotoxigenic *E. coli* (ETEC) vaccine was constructed by direct deletion of *rfbF* (36). However, our results here suggest that this mutant vaccine strain may contain secondary mutations preventing UndP in making OAg during strain construction or the *rfbF* mutation in the vaccine strain is polar to the downstream *rfbG*. In addition, OAg represents an important vaccine antigen itself when coupled with a protein carrier through bioconjugation, known as glycoconjugates (37), which was demonstrated to be effective in animals against a range of bacterial pathogens with some reaching phase two clinical trials (38). The successful production of desired bacterial polysaccharides in the engineered vaccine-producing host strain requires knowledge in understanding of the gene functions responsible for the glycan assembly. This is because it can be difficult to predict the OAg produced in strains carrying heterologous *rfb* genes. Indeed, introduction of *S. flexneri rfb* locus with *Tn* insertions in *rfbF* into *E. coli* K-12 was reported previously to produce an altered OAg type different from *S. flexneri* Y serotype (13). With the GT gene functions assigned for *S. flexneri rfb* region in this work, we predicted and confirmed that the altered OAg produced in this strain is O16 due to the functional complementation of *wbbL::IS5* by *rfbG*. Therefore, our approach paves the way for expanding the knowledge required for the genetic construction of glycoconjugate vaccine strains.

Determination of OAg GT functions in *E. coli* using a genetic approach has been reported previously (39). In the previous work, a tester strain was developed with all OAg, ECA, and colanic acid synthesis genes deleted to eliminate any potential heterologous sugar structure modifications and with the IT expression under tight control. The *rfb* regions from the target strain with putative GTs were cloned in a plasmid and introduced into the tester strain, allowing the analysis of the mass of the intermediates to infer GT functions. However, this approach requires substantial work in the construction of the tester strain and the assembly of *rfb* locus (~10 kb) of the target strain into a plasmid. In addition, the approach solely relies on the analysis of the mass of the intermediates when GTs are mutated. However, the copy number of the *wzx* genes introduced into the tester strain via plasmid could influence the OAg intermediate-linked LPS produced. This is because increased Wzx expression could have phenotypically lessened specificity (19), potentially accommodating even earlier steps of incomplete OAg precursors (which are not stalled in the synthesis) to be flipped across the IM, complicating the interpretation of results. In contrast, our approach to GT function determination was done in the parental strain background, with no changes in gene copies and expression levels for *wzx*.

A feedback mechanism in controlling the assembly of OAg RU in the cytosolic face of IM beyond the committed step is lacking, building dead-end intermediates when late GTs were mutated triggering lethal phenotype (23). Harnessing this knowledge, we innovatively demonstrated a simple genetic approach by just comparing the lethal phenotypes of mutants with each putative GT mutated to determine the second GT function. The lack of the feedback mechanism in the assembly of polysaccharides RUs seems to exist more widely and beyond OAg synthesis pathways, as shown for the ECA biosynthesis pathway (40), teichoic acid synthesis pathway in *Staphylococcus aureus* (41), and capsular polysaccharide synthesis pathways in other Gram-negative bacteria (42, 43) and Gram-positive bacteria (35, 44), the latter also representing diverse major polysaccharide antigens important for epidemiology and clinical serology. Therefore, our genetic approach for distinguishing the committed steps of putative GT between the late GTs, in principle, can be extended also to other diverse polysaccharide types as well as to other bacteria including Gram-positives.

## MATERIALS AND METHODS

### Bacterial strains and plasmids

The bacterial strains and plasmids used in this work are listed in Table 1. Single colonies of bacterial strains grown overnight on Lysogeny Broth (LB)-Lennox (45) agar (1.5% wt/vol) plates were picked and grown overnight in LB at 37°C for subsequent experiments. Where appropriate, media were supplemented with ampicillin (Amp, 100 µg/mL), kanamycin (Kan, 50 µg/mL), chloramphenicol (Chl, 25 µg/mL), anhydrotetracycline (AhTet 50 ng/mL), or arabinose (Ara, 10 mM).

**TABLE 1:** Strains, plasmids, and oligonucleotides

Strain or plasmid	Description or sequence^*a*^	Source
Bacterial strains		
MG1655	Wild-type *E. coli* K-12 MG1655	Lab stock
MG1655-S	MG1655 with IS5I removed in *wbbL*	(22)
MG1655-S*ΔwecA*	MG1655-S*ΔwecA::kan,* used for cloning	(22)
TOP10	F–mcrA Δ(mrr-hsdRMS-mcrBC) φ80lacZΔM15 ΔlacX74 recA1 araD139 Δ(ara-leu)7697 galU galK λ–rpsL(StrR) endA1 nupG	Invitrogen
PE860	*S. flexneri* Y serotype	(46)
PE860*ΔwecA*	PE860*ΔwecA::kan*	This work
PE860*ΔwecA*	PE860*ΔwecA::frt*	This work
PE860*ΔwecAΔrfbF*	PE860*ΔwecA::kan ΔrfbF::chl*	This work
PE860*ΔwecAΔrfbG*	PE860*ΔwecA::frt ΔrfbG::kan*	This work
PE860*ΔwecAΔwzxB*	PE860*ΔwecA::kan ΔwzxB::chl*	This work
Plasmids		
pSU2718	Cloning plasmid, lac promoter, Chl^R^	(47)
pBAD322G	Cloning plasmid, arabinose promoter, Gent^R^	(48)
pKD46	Temperature sensitive plasmid expressing Red proteins, Amp^R^	(49)
pCP20	Plasmid expressing FLP flippase, Amp^R^	(49)
pKD4	Plasmid carrying FRT flanked kanamycin resistant cassette, Amp^R^, Kan^R^	(49)
pKD3	Plasmid carrying FRT flanked kanamycin resistant cassette, Amp^R^, Chl^R^	(49)
pWQ572	Tetracycline inducible promoter, Chl^R^	(50)
pWbbL	*wbbL* CDS cloned from WG1 into pWQ572	(51)
pWecA	*wecA* CDS cloned from MG1655 into pBAD322G	This work
pRfbF	*rfbF* CDS cloned from PE860 into pSU2718	This work
pRfbG	*rfbG* CDS cloned from PE860 into pSU2718	This work
Oligonucleotides		
*wecA* KO F	TCGGTTTACGCAGGGATTTGCTTCACGTTCGGAATTGTCGGTGTAGGCTGGAGCTGCTTC	
*wecA* KO R	CTGCGTTTTACGCGCTTAATAAAGCGAGCAACTTTCCAGGATGGGAATTAGCCATGGTCC	
*wecA* cloning F	AATTGAATTCGTGAATTTACTGACAGTGAGTACTG	
*wecA* cloning R	AATTGGTACCTTATTTGGTTAAATTGGGGCTGC	
*wzx* KO F	GTCAAAAATTTGTAGATGCTGATTATTTTATATGATAAAGAAATGTAATAATGGGAATTAGCCATGGTCC	
*wzx* KO R	AGCGTAAATATTACTATTCATTATCCAAGTGACTCAGTAATTGGTTAATTGTGTAGGCTGGAGCTGCTTC	
*rfbF* KO F	TGTTCTGTATATGAAAACTAAAATTAACCAATTACTGAGTCACTTGGATAATGGGAATTAGCCATGGTCC	
*rfbF* KO R	TTTTTCATATATTCAATTTTATTTTTTTGAGTTGCGATAATTAAAGTCGTGTGTAGGCTGGAGCTGCTTC	
*rfbF* cloning F	AATTGGTACCATGAATAGTAATATTTACGCTGTCATTG	
*rfbF* cloning R	AATTGGATCCCTATGCATTATAACGACCGC	
*rfbG* KO F	GGGCGGTCGTTATAATGCATAGTTCTGATCAAAAAAGAGTAGCTGTACTTATGGGAATTAGCCATGGTCC	
*rfbG* KO R	ATGTTATAAAAATTTTATTTATATTATTCATATTCGTAAGGTGATGTTTTGTGTAGGCTGGAGCTGCTTC	
*rfbG* cloning F	AAATTGGTACCATGCATAGTTCTGATCAAAAAAGAGTAG	
*rfbG* cloning R	AATTGGATCCTTAAAATATGTTTAAAATCAACATTAATAAAATATAAATTTTATCAGC	

^*a*^ The underlined nucleotide sequences denote engineered restriction enzyme sites.

### Bacterial mutagenesis via allelic exchange

Mutagenesis was performed as described previously (49) with laboratory-adapted optimizations (52). Briefly *S. flexneri* strains harboring plasmid pKD46 grown overnight in 10 mL LB at 30°C was sub-cultured 1 in 100 into 10 mL LB in a 50 mL tube. Expression of the lambda phage-derived Red proteins was then induced with 50 mM L-arabinose at optical density at 600 nm (OD_600_) of 0.3 for 1 h. Bacterial cells were then harvested via centrifugation, washed twice with 10 mL ice-cold water, and resuspended in 100 µL of 10% (vol/vol) ice-cold glycerol for subsequent electroporation. The *cat* or *neo* gene was PCR-amplified from pKD3 or PKD4, respectively, with primers containing 50 bp up- and down-stream sequences homologous to the target gene (Table 1). The PCR amplicon was purified (1.5 µg) and introduced into electrocompetent cells via electroporation, and the cells were immediately recovered in 3 mL LB in a 50 mL Falcon tube that was incubated for 2 h at 37°C before plating out (100 µL) on LB agar plates supplemented with Chl or Kan. Plates were incubated at 37°C for 16 h to acquire mutants. Successful mutants were PCR screened and confirmed.

### Plasmid construction

For generation of expression constructs, the coding sequences of WecA, RfbF, and RfbG were PCR amplified from boiled whole-cell water preparation of PE860 and cloned into pBAD322G (for WecA) and pSU2718 (for RfbF and RfbG) using restriction enzyme cloning, resulting in pWecA, pRfbF, and pRfbG, respectively. RfbF and RfbG clones were recovered from MG1655-SΔ*wecA* to avoid potential OAg intermediate build-up in cloning strains.

### Bacterial survival spotting assay

Bacterial survival spotting assays were performed as described previously (22). Briefly, overnight bacterial cultures were adjusted to OD_600_ of 1-fold and 10-fold serial diluted to 10^−7^ with fresh LB media. A 4 µL of each dilution preparation was spotted onto LB agar plates supplemented with or without 10 mM arabinose.

### Bacterial growth kinetic assay

Bacterial growth kinetics were recorded as described previously (22). Briefly, overnight bacterial cultures were diluted 1 in 200 µL of fresh LB media, supplemented with or without 10 mM arabinose and/or 50 ng/mL anhydrotetracycline in a 96-well plate. Plates were incubated at 37°C with aeration in a CLARIOstar plate reader (BMG, Australia) programmed to measure the OD_600_ every 6 min over 18 h.

### Bacterial cell lysis assay

For measuring bacterial cell lysis, WecA and WbbL were induced in bacterial cells grown at OD_600_ of 1 and allowed for further incubation of 30 min at 37°C. Bacterial cultures with reduced turbidity due to cell lysis were imaged. Culture supernatants were then harvested via centrifugation (20,000 × *g*) and mixed with 5 µg/mL Ethidium bromide (EtBr, BioRad). Fluorescence was measured in a CLARIOstar plate reader (BMG, Australia) at the excitation and emission wavelength of 525 nm and 615 nm, respectively.

### LPS silver staining

LPS silver staining was performed as described previously (22). Briefly, bacterial cells (10^9^) grown at mid-exponential phase were collected via centrifugation (20,000 g, 1 min) and lysed in 50 µL of SDS sample buffer and heated at 100°C for 10 min. Samples were then cooled and treated with 50 µg/mL proteinase K (PK, NEB) for 18 h at 60°C. PK-treated samples were then heated at 100°C for 10 min; 2–5 μL samples were loaded onto 10%–20% SDS-tricine gels (Invitrogen, #EC66252BOX); and LPS was silver stained as described previously (53). For bacterial strains producing OAg intermediates substituted LPS, cells were grown to OD_600_ of 0.8 in LB with 0.2% (wt/vol) glucose, followed by washing with fresh LB media two times, then induced with 10 mM arabinose and/or 50 ng/mL anhydrotetracycline for 20 min to allow early cell lysis to occur. LPS samples were then prepared and analyzed as above.

### Western immunoblotting

For western immunoblotting of O16 LPS, polysaccharide samples separated by SDS-tricine gel electrophoresis were transferred onto nitrocellulose membrane and detected with rabbit polyclonal anti-O16 antibodies (SSI Diagnostica, #SSI85012).

### Protein sequence alignment and structure comparison analysis

Protein sequences and AlphaFold (33) predicted structures of WbbL (P36667), RfbG (P37783), and RfbF (P37782) were acquired from UniProtKB/Swiss-Prot database. Protein sequences were aligned via CLUSTAL multiple sequence alignment (Clustal Omega, EMBL’s European Bioinformatics Institute). AlphaFold-predicted protein structures were aligned using UCSF Chimera X (54) via MatchMaker. The overall alignment score and the root mean square deviation of atom pairs were reported.

## Data Availability

All data generated or analyzed during this study are included in this article.

## References

[1] Chaput C, Spindler E, Gill RT, Zychlinsky A. 2013. O-antigen protects Gram-negative bacteria from histone killing. PLoS One 8:e71097. doi:10.1371/journal.pone.0071097 23951089 PMC3738592

[2] Phan M-D, Peters KM, Sarkar S, Lukowski SW, Allsopp LP, Gomes Moriel D, Achard MES, Totsika M, Marshall VM, Upton M, Beatson SA, Schembri MA. 2013. The serum resistome of a globally disseminated multidrug resistant uropathogenic Escherichia coli clone. PLoS Genet 9:e1003834. doi:10.1371/journal.pgen.1003834 24098145 PMC3789825

[3] Ascari A, Waters JK, Morona R, Eijkelkamp BA. 2023. Shigella flexneri adapts to niche-specific stresses through modifications in cell envelope composition and decoration. ACS Infect Dis 9:1610–1621. doi:10.1021/acsinfecdis.3c00210 37494550

[4] Smith HW. 1975. Survival of orally administered E. coli K 12 in alimentary tract of man. Nature 255:500–502. doi:10.1038/255500a0 1094297

[5] Day CJ, Tran EN, Semchenko EA, Tram G, Hartley-Tassell LE, Ng PSK, King RM, Ulanovsky R, McAtamney S, Apicella MA, Tiralongo J, Morona R, Korolik V, Jennings MP. 2015. Glycan:glycan interactions: high affinity biomolecular interactions that can mediate binding of pathogenic bacteria to host cells. Proc Natl Acad Sci U S A 112:E7266–75. doi:10.1073/pnas.1421082112 26676578 PMC4702957

[6] Tran ENH, Day CJ, McCartney E, Poole J, Tse E, Jennings MP, Morona R. 2020. Shigella flexneri targets human colonic goblet cells by O antigen binding to Sialyl-Tn and Tn antigens via glycan-glycan interactions. ACS Infect Dis 6:2604–2615. doi:10.1021/acsinfecdis.0c00178 32926786

[7] West NP, Sansonetti P, Mounier J, Exley RM, Parsot C, Guadagnini S, Prévost M-C, Prochnicka-Chalufour A, Delepierre M, Tanguy M, Tang CM. 2005. Optimization of virulence functions through glucosylation of Shigella LPS. Science 307:1313–1317. doi:10.1126/science.1108472 15731456

[8] Van den Bosch L, Manning PA, Morona R. 1997. Regulation of O-antigen chain length is required for Shigella flexneri virulence. Mol Microbiol 23:765–775. doi:10.1046/j.1365-2958.1997.2541625.x 9157247

[9] Liu B, Furevi A, Perepelov AV, Guo X, Cao H, Wang Q, Reeves PR, Knirel YA, Wang L, Widmalm G. 2020. Structure and genetics of Escherichia coli O antigens. FEMS Microbiol Rev 44:655–683. doi:10.1093/femsre/fuz028 31778182 PMC7685785

[10] KAUFFMANN F. 1947. The Serology of the coli group. J Immunol 57:71–100. doi:10.4049/jimmunol.57.1.71 20264689

[11] Larsson P, Andersson HE, Norlén B. 1981. Epidemiological tracing of Escherichia coli by O antigen typing in a geriatric ward. Scand J Infect Dis 13:185–190. doi:10.3109/inf.1981.13.issue-3.05 7031857

[12] Samuel G, Reeves P. 2003. Biosynthesis of O-antigens: genes and pathways involved in nucleotide sugar precursor synthesis and O-antigen assembly. Carbohydr Res 338:2503–2519. doi:10.1016/j.carres.2003.07.009 14670712

[13] Morona R, Macpherson DF, Van Den Bosch L, Carlin NI, Manning PA. 1995. Lipopolysaccharide with an altered O-antigen produced in Escherichia coli K-12 harbouring mutated, cloned Shigella flexneri rfb genes. Mol Microbiol 18:209–223. doi:10.1111/j.1365-2958.1995.mmi_18020209.x 8709841

[14] Lairson LL, Henrissat B, Davies GJ, Withers SG. 2008. Glycosyltransferases: structures, functions, and mechanisms. Annu Rev Biochem 77:521–555. doi:10.1146/annurev.biochem.76.061005.092322 18518825

[15] Wang W, Perepelov AV, Feng L, Shevelev SD, Wang Q, Senchenkova SN, Han W, Li Y, Shashkov AS, Knirel YA, Reeves PR, Wang L. 2007. A group of Escherichia coli and Salmonella *e*nterica O antigens sharing a common backbone structure. Microbiology (Reading) 153:2159–2167. doi:10.1099/mic.0.2007/004192-0 17600060

[16] Rush JS, Alaimo C, Robbiani R, Wacker M, Waechter CJ. 2010. A novel epimerase that converts GlcNAc-P-P-undecaprenol to GalNAc-P-P-undecaprenol in Escherichia coli O157. J Biol Chem 285:1671–1680. doi:10.1074/jbc.M109.061630 19923219 PMC2804325

[17] Liu D, Cole RA, Reeves PR. 1996. An O-antigen processing function for Wzx (RfbX): a promising candidate for O-unit flippase. J Bacteriol 178:2102–2107. doi:10.1128/jb.178.7.2102-2107.1996 8606190 PMC177911

[18] Rick PD, Barr K, Sankaran K, Kajimura J, Rush JS, Waechter CJ. 2003. Evidence that the wzxE gene of Escherichia coli K-12 encodes a protein involved in the transbilayer movement of a trisaccharide-lipid intermediate in the assembly of enterobacterial common antigen. J Biol Chem 278:16534–16542. doi:10.1074/jbc.M301750200 12621029

[19] Liu MA, Stent TL, Hong Y, Reeves PR. 2015. Inefficient translocation of a truncated O unit by a Salmonella Wzx affects both O-antigen production and cell growth. FEMS Microbiol Lett 362:fnv053. doi:10.1093/femsle/fnv053 25837817

[20] Hong Y, Cunneen MM, Reeves PR. 2012. The Wzx translocases for Salmonella enterica O-antigen processing have unexpected serotype specificity. Mol Microbiol 84:620–630. doi:10.1111/j.1365-2958.2012.08048.x 22497246

[21] Schmidt G. 1973. Genetical studies on the lipopolysaccharide structure of Escherichia coli K12. J Gen Microbiol 77:151–160. doi:10.1099/00221287-77-1-151 4579437

[22] Qin J, Hong Y, Morona R, Totsika M. 2023. O antigen biogenesis sensitises Escherichia coli K-12 to bile salts, providing a plausible explanation for its evolutionary loss. PLoS Genet 19:e1010996. doi:10.1371/journal.pgen.1010996 37792901 PMC10578602

[23] Hong Y, Reeves PR. 2016. Model for the controlled synthesis of O-antigen repeat units involving the WaaL Ligase. mSphere 1:e00074-15. doi:10.1128/mSphere.00074-15 PMC4863624 27303678

[24] van Heijenoort J. 2001. Formation of the glycan chains in the synthesis of bacterial peptidoglycan. Glycobiology 11:25R–36R. doi:10.1093/glycob/11.3.25r 11320055

[25] Kenne L, Lindberg B, Petersson K. 1977. Basic structure of the oligosaccharide repeating-unit of the Shigella flexneri O-antigens. Carbohydr Res 56:363–370. doi:10.1016/s0008-6215(00)83357-1 332361

[26] Formal SB, Gemski P, Baron LS, Labrec EH. 1970. Genetic transfer of Shigella flexneri antigens to Escherichia coli K-12. Infect Immun 1:279–287. doi:10.1128/iai.1.3.279-287.1970 16557729 PMC415893

[27] Liu B, Knirel YA, Feng L, Perepelov AV, Senchenkova SN, Wang Q, Reeves PR, Wang L. 2008. Structure and genetics of Shigella O antigens. FEMS Microbiol Rev 32:627–653. doi:10.1111/j.1574-6976.2008.00114.x 18422615

[28] Reeves PR, Hobbs M, Valvano MA, Skurnik M, Whitfield C, Coplin D, Kido N, Klena J, Maskell D, Raetz CR, Rick PD. 1996. Bacterial polysaccharide synthesis and gene nomenclature. Trends Microbiol 4:495–503. doi:10.1016/s0966-842x(97)82912-5 9004408

[29] Liu D, Reeves PR. 1994. Escherichia coli K12 regains its O antigen. Microbiology (Reading) 140 ( Pt 1):49–57. doi:10.1099/13500872-140-1-49 7512872

[30] Stevenson G, Neal B, Liu D, Hobbs M, Packer NH, Batley M, Redmond JW, Lindquist L, Reeves P. 1994. Structure of the O antigen of Escherichia coli K-12 and the sequence of its rfb gene cluster. J Bacteriol 176:4144–4156. doi:10.1128/jb.176.13.4144-4156.1994 7517391 PMC205614

[31] Nath P, Tran ENH, Morona R. 2015. Mutational analysis of the Shigella flexneri O-antigen polymerase Wzy: identification of Wzz-dependent Wzy mutants. J Bacteriol 197:108–119. doi:10.1128/JB.01885-14 25313393 PMC4288684

[32] Hu Y, Walker S. 2002. Remarkable structural similarities between diverse glycosyltransferases. Chem Biol 9:1287–1296. doi:10.1016/s1074-5521(02)00295-8 12498881

[33] Jumper J, Evans R, Pritzel A, Green T, Figurnov M, Ronneberger O, Tunyasuvunakool K, Bates R, Žídek A, Potapenko A, et al.. 2021. Highly accurate protein structure prediction with AlphaFold. Nature 596:583–589. doi:10.1038/s41586-021-03819-2 34265844 PMC8371605

[34] James DBA, Gupta K, Hauser JR, Yother J. 2013. Biochemical activities of Streptococcus pneumoniae serotype 2 capsular glycosyltransferases and significance of suppressor mutations affecting the initiating glycosyltransferase Cps2E. J Bacteriol 195:5469–5478. doi:10.1128/JB.00715-13 24097952 PMC3889599

[35] Xayarath B, Yother J. 2007. Mutations blocking side chain assembly, polymerization, or transport of a Wzy-dependent Streptococcus pneumoniae capsule are lethal in the absence of suppressor mutations and can affect polymer transfer to the cell wall. J Bacteriol 189:3369–3381. doi:10.1128/JB.01938-06 17322316 PMC1855910

[36] Girardi P, Harutyunyan S, Neuhauser I, Glaninger K, Korda O, Nagy G, Nagy E, Szijártó V, Pall D, Szarka K, Kardos G, Henics T, Malinoski FJ. 2022. Evaluation of the safety, tolerability and immunogenicity of shigetec an oral live attenuated Shigella-ETEC vaccine in placebo-controlled randomized phase 1 trial. Vaccines (Basel) 10:340. doi:10.3390/vaccines10020340 35214798 PMC8879453

[37] Feldman MF, Wacker M, Hernandez M, Hitchen PG, Marolda CL, Kowarik M, Morris HR, Dell A, Valvano MA, Aebi M. 2005. Engineering N-linked protein glycosylation with diverse O antigen lipopolysaccharide structures in Escherichia coli. Proc Natl Acad Sci U S A 102:3016–3021. doi:10.1073/pnas.0500044102 15703289 PMC549450

[38] Dow JM, Mauri M, Scott TA, Wren BW. 2020. Improving protein glycan coupling technology (PGCT) for glycoconjugate vaccine production. Expert Rev Vaccines 19:507–527. doi:10.1080/14760584.2020.1775077 32627609

[39] Stevenson G, Dieckelmann M, Reeves PR. 2008. Determination of glycosyltransferase specificities for the Escherichia coli O111 O antigen by a generic approach. Appl Environ Microbiol 74:1294–1298. doi:10.1128/AEM.02660-07 18156323 PMC2258590

[40] Jorgenson MA, Kannan S, Laubacher ME, Young KD. 2016. Dead-end intermediates in the enterobacterial common antigen pathway induce morphological defects in Escherichia coli by competing for undecaprenyl phosphate. Mol Microbiol 100:1–14. doi:10.1111/mmi.13284 26593043 PMC4845916

[41] D’Elia MA, Pereira MP, Chung YS, Zhao W, Chau A, Kenney TJ, Sulavik MC, Black TA, Brown ED. 2006. Lesions in teichoic acid biosynthesis in Staphylococcus aureus lead to a lethal gain of function in the otherwise dispensable pathway. J Bacteriol 188:4183–4189. doi:10.1128/JB.00197-06 16740924 PMC1482942

[42] Tan YH, Chen Y, Chu WHW, Sham LT, Gan YH. 2020. Cell envelope defects of different capsule-null mutants in K1 hypervirulent Klebsiella pneumoniae can affect bacterial pathogenesis. Mol Microbiol 113:889–905. doi:10.1111/mmi.14447 31912541 PMC7317392

[43] Bai J, Dai Y, Farinha A, Tang AY, Syal S, Vargas-Cuebas G, van Opijnen T, Isberg RR, Geisinger E. 2021. Essential gene analysis in Acinetobacter baumannii by high-density transposon mutagenesis and CRISPR interference. J Bacteriol 203:e0056520. doi:10.1128/JB.00565-20 33782056 PMC8316057

[44] Nakamoto R, Kwan JMC, Chin JFL, Ong HT, Flores-Kim J, Midonet C, VanNieuwenhze MS, Guan XL, Sham LT. 2021. The bacterial tyrosine kinase system CpsBCD governs the length of capsule polymers. Proc Natl Acad Sci U S A 118:e2103377118. doi:10.1073/pnas.2103377118 34732571 PMC8609450

[45] LENNOX ES. 1955. Transduction of linked genetic characters of the host by bacteriophage P1. Virology 1:190–206. doi:10.1016/0042-6822(55)90016-7 13267987

[46] Maczuga N, Tran ENH, Qin J, Morona R. 2022. Interdependence of Shigella flexneri O antigen and enterobacterial common antigen biosynthetic pathways. J Bacteriol 204:e0054621. doi:10.1128/jb.00546-21 35293778 PMC9017295

[47] Martinez E, Bartolomé B, de la Cruz F. 1988. pACYC184-derived cloning vectors containing the multiple cloning site and lacZ alpha reporter gene of pUC8/9 and pUC18/19 plasmids. Gene 68:159–162. doi:10.1016/0378-1119(88)90608-7 2851489

[48] Cronan JE. 2006. A family of arabinose-inducible Escherichia coli expression vectors having pBR322 copy control. Plasmid 55:152–157. doi:10.1016/j.plasmid.2005.07.001 16139359

[49] Datsenko KA, Wanner BL. 2000. One-step inactivation of chromosomal genes in Escherichia coli K-12 using PCR products. Proc Natl Acad Sci U S A 97:6640–6645. doi:10.1073/pnas.120163297 10829079 PMC18686

[50] Larue K, Ford RC, Willis LM, Whitfield C. 2011. Functional and structural characterization of polysaccharide co-polymerase proteins required for polymer export in ATP-binding cassette transporter-dependent capsule biosynthesis pathways. J Biol Chem 286:16658–16668. doi:10.1074/jbc.M111.228221 21454677 PMC3089508

[51] Hong Y, Reeves PR. 2014. Diversity of O-antigen repeat unit structures can account for the substantial sequence variation of wzx translocases. J Bacteriol 196:1713–1722. doi:10.1128/JB.01323-13 24532778 PMC3993327

[52] Qin J, Hong Y, Pullela K, Morona R, Henderson IR, Totsika M. 2022. A method for increasing electroporation competence of gram-negative clinical isolates by polymyxin B nonapeptide. Sci Rep 12:11629. doi:10.1038/s41598-022-15997-8 35804085 PMC9270391

[53] Tsai CM, Frasch CE. 1982. A sensitive silver stain for detecting lipopolysaccharides in polyacrylamide gels. Anal Biochem 119:115–119. doi:10.1016/0003-2697(82)90673-x 6176137

[54] Meng EC, Goddard TD, Pettersen EF, Couch GS, Pearson ZJ, Morris JH, Ferrin TE. 2023. UCSF chimerax: tools for structure building and analysis. Protein Sci 32:e4792. doi:10.1002/pro.4792 37774136 PMC10588335

